# Loss of autophagy in dopaminergic neurons causes Lewy pathology and motor dysfunction in aged mice

**DOI:** 10.1038/s41598-018-21325-w

**Published:** 2018-02-12

**Authors:** Shigeto Sato, Toshiki Uchihara, Takahiro Fukuda, Sachiko Noda, Hiromi Kondo, Shinji Saiki, Masaaki Komatsu, Yasuo Uchiyama, Keiji Tanaka, Nobutaka Hattori

**Affiliations:** 1 Department of Neurology, Juntendo University Graduate School of Medicine, Tokyo, 113-8421 Japan; 2 Laboratory of Structural Neuropathology, Tokyo Metropolitan Institute of Medical Science, Tokyo, 156-8506 Japan; 3 Division of Neuropathology, Department of Neuropathology, The Jikei University, School of Medicine, Tokyo, 105-8461 Japan; 4 Histology Center, Tokyo Metropolitan Institute of Medical Science, Tokyo, 156-8506 Japan; 5 Department of Biochemistry, Niigata University Graduate School of Medical and Dental Sciences, Chuo-ku, Niigata 951–8510 Japan; 6 Department of Cellular and Molecular Neuropathology, Juntendo University Graduate School of Medicine, Tokyo, 113-8421 Japan; 7 Laboratory of Protein Metabolism, Tokyo Metropolitan Institute of Medical Science, Tokyo, 156-8506 Japan

## Abstract

Inactivation of constitutive autophagy results in the formation of cytoplasmic inclusions in neurons, but the relationship between impaired autophagy and Lewy bodies (LBs) as well as the *in vivo* process of formation remains unknown. Synuclein, a component of LBs, is the defining characteristic of Parkinson’s disease (PD). Here, we characterize dopamine (DA) neuron–specific autophagy-deficient mice and provide *in vivo* evidence for LB formation. Synuclein deposition is preceded by p62 and resulted in the formation of inclusions containing synuclein and p62. The number and size of these inclusions were gradually increased in neurites rather than soma with aging. These inclusions may facilitate peripheral failures. As a result, DA neuron loss and motor dysfunction including the hindlimb defect were observed in 120-week-old mice. P62 aggregates derived from an autophagic defect might serve as “seeds” and can potentially be cause of LB formation.

## Introduction

Parkinson’s disease (PD), the most common neurodegenerative disorder, is characterized by the loss of nigrostriatal dopamine neurons and the formation of intracellular Lewy bodies (LBs), which consist primarily of α-synuclein (hereafter referred to as synuclein) and ubiquitin^1^. Interactions between genetic predisposition and environmental factors are likely the primary events inducing mitochondrial dysfunction and oxidative damage, resulting in oligomerization and aggregation of synuclein, but the underlying molecular mechanisms remain poorly understood. The vast majority of PD occurs sporadically, with inherited familial forms of the disease accounting for roughly 5% of all cases^2^. The identification of PD-related genes and risk factors has implicated several pathways in PD etiology, with growing evidence suggesting a link between dysfunctional intracellular protein catabolism and PD pathogenesis. In *PARK1*-linked PD, intrinsically disordered mutant synuclein initiates the disease process. Given that highly aggregated proteins are deposited in nigral neurons in PD, dysfunctions of proteolytic systems, i.e., the ubiquitin–proteasome system and autophagy–lysosomal pathway, seem to contribute to the final neurodegenerative process.

Macroautophagy (hereafter, referred to as autophagy) is a highly conserved bulk protein degradation pathway in eukaryotes. Cytoplasmic proteins and organelles are engulfed within autophagosomes, which fuse with the lysosome, where they are degraded along with their cargo^3^. Several lines of evidence indicate that synuclein is predominantly degraded by autophagy, but also by the proteasome. However, mutant forms of synuclein and oligomers are dependent on the autophagy–lysosome pathway for their clearance^4–6^. Although the phenotypes of mice harboring brain-specific deletion of *Atg5* or *Atg7* reveal the critical role of autophagy in the removal of aggregated proteins^7,8^, Friedman *et al*.^9^ demonstrated that DA neuron-specific autophagy deficiency leads to the restrictive presynaptic accumulation of synuclein in the dorsal striatum, suggesting that impaired autophagy plays a role in PD pathogenesis. In DA neurons, the primary site of endogenous pathology, no detailed reports to date have examined the endogenous synuclein accumulation in dopaminergic cell bodies and neurites, which is associated with Lewy pathology. In this context, the stress-inducible protein p62 (encoded by the gene Sqstm1) is of importance because it is the ubiquitin- and LC3-binding protein that functions as a selective autophagy adaptor/receptor for degradation of ubiquitinated substrates^10^. Indeed, p62 is present in neuronal inclusions of individuals with Alzheimer’s disease and other neurodegenerative diseases^11^, although its actual roles in pathogenesis remains largely unknown.

In order to understand the effects of autophagy impairment on DA neurons, we characterized conditional knock-out mice harboring a tyrosine hydroxylase (TH) neuron–specific deletion of *Atg7* and observed their age-related pathological and motor phenotypes.

## Results

### Generation of DA neuron–specific *Atg7* conditional knockout mice, and characterization of their locomotor impairments

**Figure 1:**
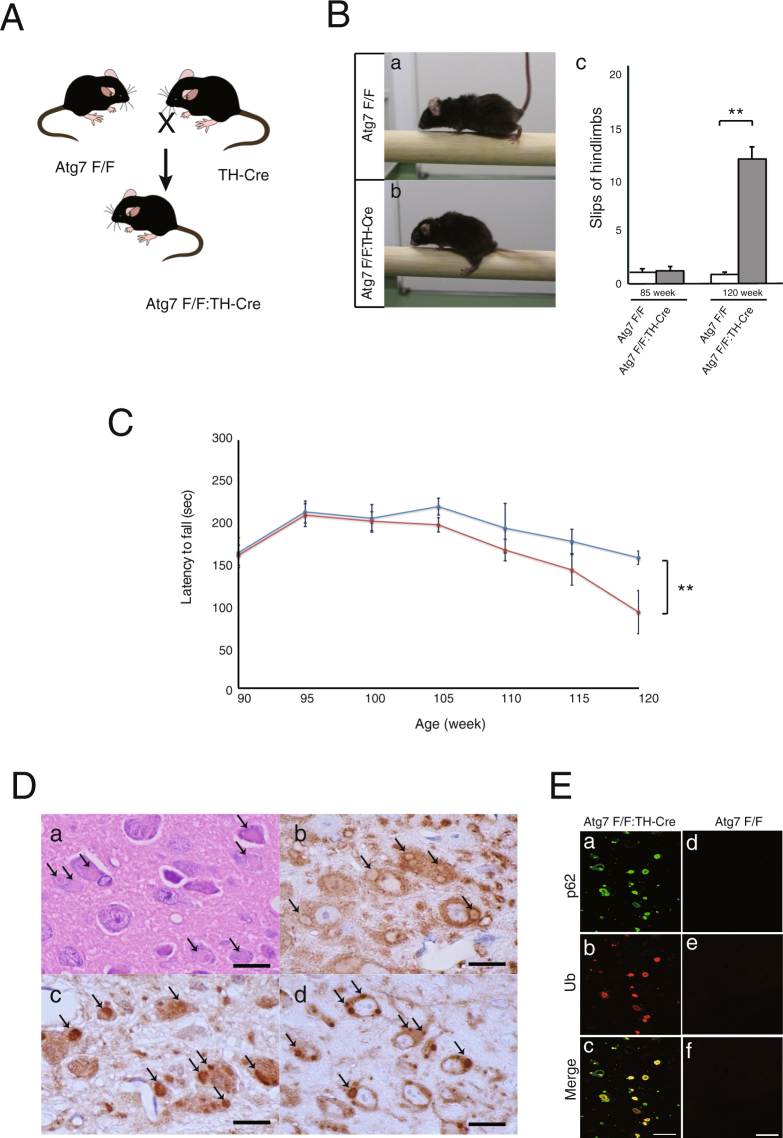
Behavioral and neuropathological examinations of *Atg7*^*flox*/*flox*^:TH-Cre mice. (**A**) Schematic representation of the genetic cross between mice carrying the floxed *Atg7* allele (*Atg7* F/F) and knock-in mice carrying Cre inserted at the 3’UTR of the TH gene (TH-Cre) to generate *Atg7* F/F:TH-Cre (or *Atg7*^*flox*/*flox*^:TH-Cre) mice. (**B**) Runway test of *Atg7*^*flox*/*flox*^ mice (a) and *Atg7*^*flox*/*flox*^:TH-Cre mice. (b) In contrast to the *Atg7*^*flox*/*flox*^ mice, which exhibited well-coordinated movement and almost no slips of the forepaw or hindpaw from the beam, *Atg7*^*flox*/*flox*^:TH-Cre mice could hardly move on the beam and the hindpaw slipped frequently (over 110 weeks of age). In this figure, the hindpaw of the *Atg7*^*flox*/*flox*^:TH-Cre mice has slipped off the beam. (c) The number of hindlimb slips was recorded as 85-week-old and 120-week-old mice cross the challenging beam. Data show means ± SE (error bars), and statistical significance was evaluated using Student’s t-test. **P < 0.01. (**C**) In the accelerating rotarod assay, rotation was accelerated from 3 rpm to 35 rpm over 5 min, and fall latency was recorded, in mice from 95 to 120 weeks of age. Red line, *Atg7*^*flox*/*flox*^:TH-Cre mice; blue line, *Atg7*^*flox*/*flox*^ mice. Data show means ± SE (error bars), and statistical significance was evaluated using Student’s t-test. **P < 0.01. (**D**) Histological analyses of locus coeruleus (LC) in a 9-month-old *Atg7*^*flox*/*flox*^:TH-Cre mouse. Paraffin sections were stained with hematoxylin–eosin (HE) (a) and immunostained for TH (b), ubiquitin (c), and p62 (d). In the each staining (a-d), many inclusions are apparent (arrow). Scale bars: 10 µm. (**E**) Immunofluorescence labeling of p62 (green) or ubiquitin (red) in the LC of an *Atg7*^*flox*/*flox*^:TH-Cre mouse (a–c) and an *Atg7*^*flox*/*flox*^ mouse (d–f)., Scale bars: 20 µm.

We generated tyrosine hydroxylase (TH) cell–specific *Atg7* conditional knockout mice (*Atg7*^*flox*/*flox*^:TH-Cre) by crossing the previously characterized *Atg7* floxed mice (*Atg7*^*flox*/*flox*^)^8^ with TH-Cre mice (TH-Cre) harboring the Cre recombinase coding sequence downstream of a characterized fragment of the TH promoter^12^ instead of the IRES version (Fig. 1A). In *Atg7*^*flox*/*flox*^:TH-Cre midbrains, the quantification of Atg7 after normalization by actin (Supple. Figure 1B) showed 13.54 ± 2.27% residual amount, compared to controls. Considering the percentage of dopamine neurons is not so high, it is intriguing that the decrease in Atg7 is remarkable.

*Atg7*^*flox*/*flox*^:TH-Cre mice were viable at birth and indistinguishable in appearance from their littermates, and their survival rate was not markedly diminished. Although *Atg7*^*flox*/*flox*^:TH-Cre mice have not yet been observed over the entire lifespan, they began to show impairment in motor coordination tasks around 100 weeks and apparent motor behavioral deficits around 110 weeks. These clinical abnormalities could be demonstrated by the runway test (Fig. 1Ba,b and c) and rotarod test (Fig. 1C). In contrast to *Atg7*^*flox*/*flox*^ mice, which exhibited well-coordinated movement and almost no slips of the forepaw or hindpaw from the beam, the, *Atg7*^*flox*/*flox*^:TH-Cre mice could hardly move on the beam and slipped frequently. In particular, the hindpaws of *Atg7*^*flox*/*flox*^:TH-Cre mice often slipped off the beam (Fig. 1Bb and c). Furthermore, in the accelerating rotarod test, the fall latency was reduced in *Atg7*^*flox*/*flox*^:TH-Cre mice (Fig. 1C). Gait disturbance progressed, and by the terminal stage, the majority of affected mice could hardly move.

### Loss of *Atg7* leads to age-related development of p62 inclusions in the DA neuron

**Figure 2:**
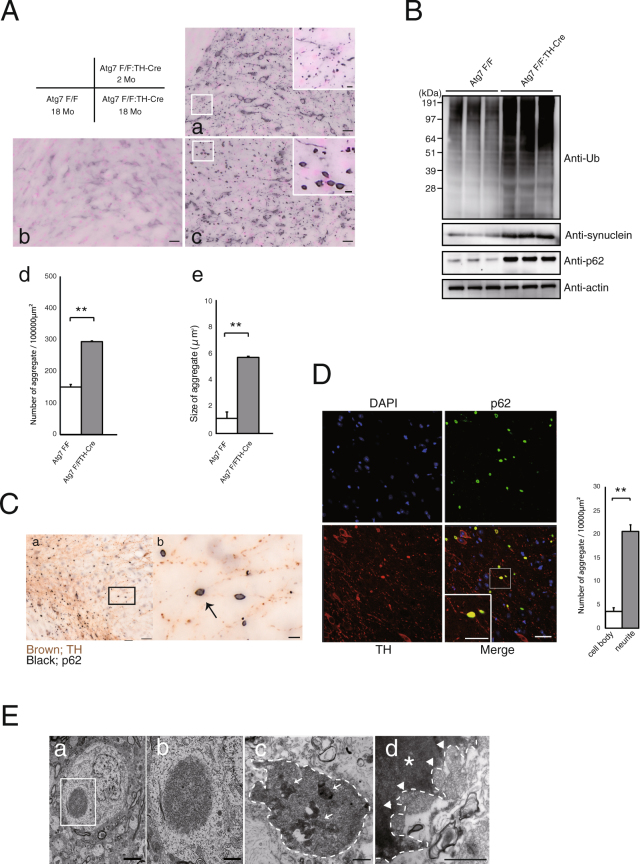
Development of p62-positive aggregates in dopaminergic neurons. (**A**) Histological analyses of 2-month-old mice (a), 18-month-old *Atg7*^*flox*/*flox*^ control (b), and 18-month old *Atg7*^*flox*/*flox*^:TH-Cre mice (c). Cryosections were immunostained for p62. In contrast to *Atg7*^*flox*/*flox*^ mice, in which no aggregates were seen in dopaminergic neurons, p62 positive aggregates increased in number (d) and size (e) in the dopaminergic neurons of *Atg7*^*flox*/*flox*^:TH-Cre mice as a function of age. Scale bars: 20 µm. High magnification of p62 inclusions is shown in the inset. Scale bar: 50 µm (inset) Data show means ± SE (error bars), and statistical significance was evaluated using Student’s t-test. **P < 0.01. (**B**) Immunoblot for ubiquitin, synuclein, p62, and actin. Lanes 1–3: 18-month-old *Atg7*^*flox*/*flox*^ mice; lanes 4–6: 18-month-old *Atg7*^*flox*/*flox*^:TH-Cre mice. (**C**) (a) Histological analyses of dopaminergic neurons in an 18-month-old *Atg7*^*flox*/*flox*^:TH-Cre mouse. Cryosections were immunostained for p62 (black) and TH (brown) Scale bars: 50 µm. Square areas in (a) are enlarged in (b). P62 inclusion (arrow) is present along TH fibers. Scale bars: 10 µm. (**D**) The number of p62-positive aggregates within TH fibers was identified by high-resolution image through dopaminergic neurons revealed immunofluorescence labeling of TH (red) and p62 (green). Scale bars: 20 µm. High magnification of inclusion is shown in the inset. Scale bars: 20 µm (inset). Data show means ± SE (error bars), and statistical significance was evaluated using Student’s t-test. **P < 0.01. (**E**) Electron micrographs of neurons in the substantia nigra (SN). Somata (a,b) and neurites (c,d) of 18-month-old *Atg7*^*flox*/*flox*^:TH-Cre mice. Square areas in (a) are enlarged in (b). Electron-dense filamentous inclusions were observed in neurons of the SN. (c) Inclusion in a neurite, containing mitochondria and autophagosomes (white arrows). Inclusion is labeled with a dashed line. (d) Electron micrograph of an inclusion immunostained for p62. Electron-dense area (white asterisk) indicates a p62-positive aggregate, which contains many mitochondria (white arrowheads). Inclusion is labeled with a dashed line. Scale bars: (a) 5 µm, (b) 2 µm, (c) 1 µm, (d) 500 nm.

Previous studies showed that deletion of *Atg5* or *Atg7* in the central nervous system (CNS) leads to formation of inclusions positive for the autophagy adaptor/receptor protein p62 in various neuronal populations. However, the mechanisms underlying age-related progression and intracellular localization of these inclusions in dopaminergic neurons remain elusive. Initially, we confirmed aggregate formation in the locus coeruleus (LC), because the somata of neurons in the LC are wide enough to easily visualize the inclusions. In *Atg7*^*flox*/*flox*^:TH-Cre mice, these neurons contained 7 eosinophilic aggregates, which are characteristic of LBs (Fig. 1Da). In addition, these inclusions were observed in TH-positive neurons (Fig. 1Db), including ubiquitin (Fig. 1Dc) and p62 (Fig. 1Dd). In fact, p62 and ubiquitin colocalization can make a strong prediction of LB (Fig. 1E). Next, we observed the staining of dopaminergic neurons in the substantia nigra (SN). In contrast to *Atg7*^*flox*/*flox*^ mice, in which no aggregates were seen even at the age of 18 months (Fig. 2Ab), p62-positive inclusions were present in 2-month-old *Atg7*^*flox*/*flox*^:TH-Cre mice (Fig. 2Aa). Notably, Western blotting revealed p62 and ubiquitin accumulations in the midbrain of 2 month-old *Atg7*^*flox*/*flox*^:TH-Cre mice, whereas synuclein did not accumulate in the midbrain, at least at this stage (data not shown). In immunohistochemical staining, dopaminergic neurons exhibited no synuclein accumulation (data not shown). In 18-month-old mice, p62 inclusions were larger (Fig. 2Ae and B) and mainly localized outside the soma (Fig. 2Ac). To determine the location of these inclusions, we conducted double DAB staining with p62 (Fig. 2C black) and TH antibodies (Fig. 2C brown). P62 inclusions were present along the TH fibers, and were mainly located in the branch of dopaminergic neurons (Fig. 2Cb). To investigate the localizations of aggregates outside the soma, we conducted double labeling (Fig. 2D). Specifically, we labeled dopaminergic neurons with TH antibody (Fig. 2D red) and followed individual fibers, revealing that p62-positive aggregates (Fig. 2D green) were present along neurites. To further characterize these aggregates, we performed ultrastructural analysis in SN (Fig. 2E). Fibrous inclusions localized in the soma (Fig. 2Ea,b) and neurites (Fig. 2Ec,d) in DA neurons. In these inclusions, autophagosome-like structures were observed (Fig. 2Ec). But, as to autophagosome formation, ATG conjugation systems including Atg7 are not absolutely essential^13^. In fact, these autophagosome-like structures were previously reported in Atg7-deficient brains^14^.

To distinguish the p62 positive inclusions, we immunostained SN slices with p62 antibody and identified lesions based on the DAB-labeled products. Ultrathin sections containing these target lesions were cut and examined under an electron microscope. In Fig. 2Ed, the black region contains p62 positive aggregates. Because such aggregates involve many mitochondria, we speculate that mitochondrial transport might be disturbed in DA neurons if these aggregates were formed in neurites.

### P62-positive inclusions contain endogenous synuclein

**Figure 3:**
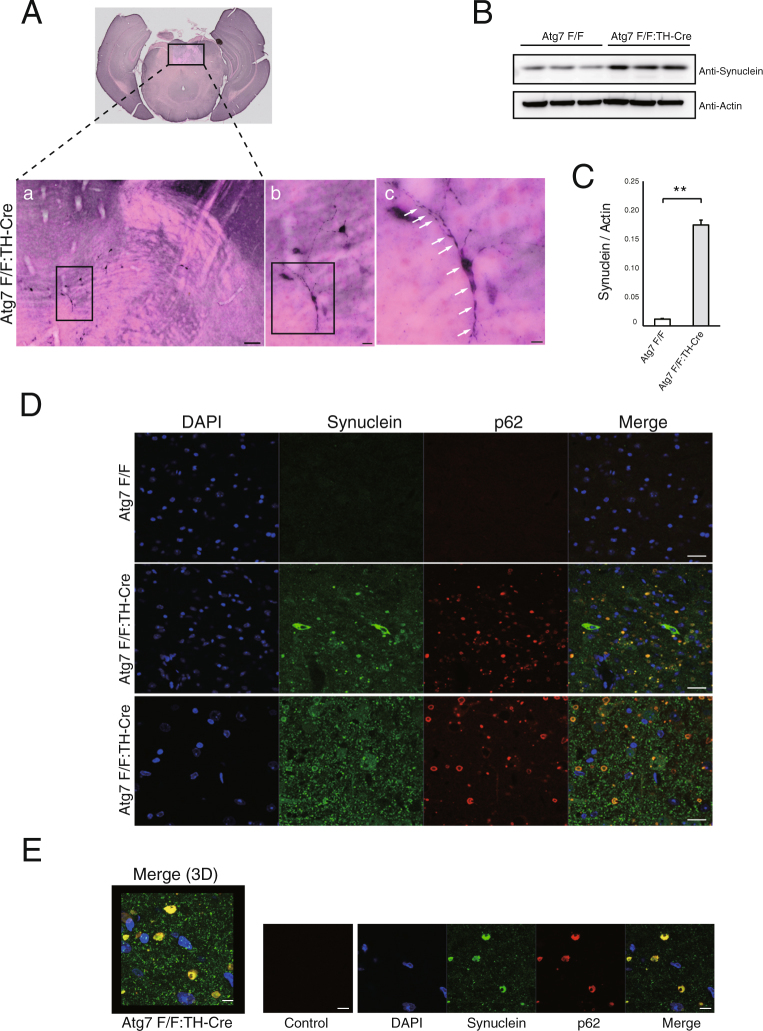
Endogenous synuclein accumulates in dopaminergic neurons of *Atg7*^*flox*/*flox*^:TH-Cre mice. (**A**) (a) Histological analyses of substantia nigra (SN) in a 9-month-old *Atg7*^*flox*/*flox*^:TH-Cre mouse. Thickly sliced (40-µm) cryosections were immunostained for synuclein, and the SN was broadly observed on a VS120 microscopic 3D measurement system (Olympus). Scale bars: 100 µm. Square areas in (a) are enlarged in (b) (Scale Bar: 20 µm) and square areas in (b) are enlarged in (c) (Scale bars: 10 µm). Arrows indicate synuclein inclusions within soma and neurites. (**B**) Immunoblot for synuclein and actin. Lanes 1–3: 9-month-old *Atg7*^*flox*/*flox*^ mouse; lanes 4–6: 9-month-old *Atg7*^*flox*/*flox*^:TH-Cre mouse. (**C**) Quantitation of the immunoblot for synuclein; ratio of synuclein to actin is shown. Synuclein accumulated in the midbrains of *Atg7*^*flox*/*flox*^:TH-Cre mice. Data show means ± SE (n = 3). **P < 0.01 (Student’s *t-*test). (**D**) Immunofluorescence labeling of synuclein (green) or p62 (red) in the SN of an *Atg7*^*flox*/*flox*^ mouse (Upper panels) and an *Atg7*^*flox*/*flox*^:TH-Cre mouse (middle and lower panels). Synuclein accumulates in somata (middle panels) and/or neurites (lower panels), where synuclein colocalizes with p62-positive inclusions. DAPI nuclear staining is shown in blue, Scale bars: 20 µm. (**E**) High-resolution confocal three-dimensional (3D) image of p62 and synuclein positive inclusions, which localize around soma in the midbrain. DAPI nuclear staining is shown in blue. Scale bars: 10 µm.

Previous *in vivo* analysis of DA neurons suggested that synuclein regulation is linked to autophagy^9,15^, but it remained unclear how synuclein accumulation is associated with PD pathology. To address this issue, we asked whether endogenous synuclein colocalizes with p62-positive inclusions in *Atg7*^*flox*/*flox*^:TH-Cre mice. To this end, we carefully observed thickly sliced midbrain blocks, which includes the SN, using a VS120 microscopic 3D measurement system (Olympus, Tokyo, Japan). Surprisingly, around 9 months, we pathologically confirmed endogenous synuclein accumulation in somata and neurites (Fig. 3A). High resolution confocal images through SN show immunofluorescence labeling of TH and synuclein, where 89.30 ± 1.65% (n = 5, each 2 slices) synuclein inclusions located in TH fibers (Supple. Figure 2C). Furthermore, the level of synuclein increased in the midbrain of *Atg7*^*flox*/*flox*^:TH-Cre mice (Fig. 3B and C). Although p62 is a representative substrate of autophagy that is rapidly influenced by its dysfunction, it seemed that not only p62, but also endogenous synuclein, was regulated to some extent by autophagy. Next, we investigated whether endogenous synuclein colocalized with p62 aggregates. In confocal microscopy, *Atg7*^*flox*/*flox*^ mice exhibited no p62-positive aggregates or endogenous synuclein accumulation (Fig. 3D upper panel). On the other hand, *Atg7*^*flox*/*flox*^:TH-Cre mice exhibited synuclein deposition and inclusions that colocalized with p62 in cell bodies (Fig. 3D middle panel). Interestingly, we observed these inclusions not only within the soma, but also outside the soma (Fig. 3D lower panel). To determine the localization of these inclusions, we analyzed many samples by confocal microscopy, and found that most inclusions localized in neurites, as demonstrated in Fig. 2D. When we observed these inclusions at higher magnification, they have multiple cavities (Fig. 3E), consistent with the results of ultrastructural analysis (Fig. 2Ed). Thus, loss of autophagy induces synuclein accumulation and formation of LBs, similar to the pathology observed in DA neurons.

### The number of TH neurons decreases in aged *Atg7*^*flox*/*flox*^:TH-Cre mice, contributing to locomotor dysfunction

**Figure 4:**
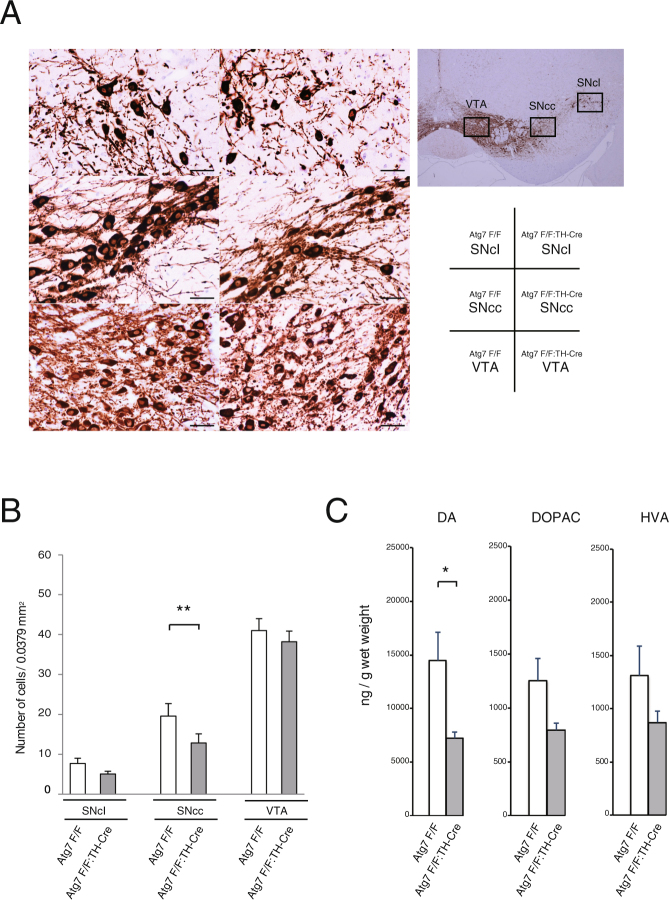
Delayed dopaminergic neuronal degeneration in *Atg7*^*flox*/*flox*^:TH-Cre mice. (**A**) Histological analyses of SN in 120-week-old *Atg7*^*flox*/*flox*^:TH-Cre and *Atg7*^*flox*/*flox*^ mice. Paraffin sections were immunostained for TH and demonstrated as shown the Table. SNcl: lateral area of substantia nigra pars compacta; SNcc: center area of substantia nigra pars compacta; VTA: Ventral Tegmental Area. Scale bars: 20 µm. (**B**) The number of dopaminergic neurons in the SN was identified by TH immunoreactivity. Data are means ± SE (*Atg7*^*flox*/*flox*^:TH-Cre, n = 4; *Atg7*^*flox*/*flox*^ mice, n = 5) **P < 0.01 (Student’s *t-*test). (**C**) HPLC analysis of DA, DOPAC, and HVA levels in the dorsal striatum at 120 weeks. Data are means ± SE (*Atg7*^*flox*/*flox*^:TH-Cre, n = 3; *Atg7*^*flox*/*flox*^ mice, n = 3) *P = 0.05 (Student’s *t-*test).

To date, many genetically modified mice have been developed as PD models, but most of them do not exhibit neuronal loss. To assess the influence of aggregate formation, we compared the number of TH neurons between aged *Atg7*^*flox*/*flox*^:TH-Cre and *Atg7*^*flox*/*flox*^ mice. As demonstrated in the rotarod test (Fig. 1C), *Atg7*^*flox*/*flox*^:TH-Cre mice exhibited locomotor dysfunction at ages above 110 weeks. We sacrificed these mice (*Atg7*^*flox*/*flox*^:TH-Cre, n = 4; *Atg7*^*flox*/*flox*^ mice, n = 5) and counted TH neurons in three sections (VTA: Ventral Tegmental Area, SNcc: center area of substantia nigra pars compacta, SNcl: lateral area of substantia nigra pars compacta) (Fig. 4A). *Atg7*^*flox*/*flox*^:TH-Cre mice had fewer TH neurons than *Atg7*^*flox*/*flox*^ mice. The reduction in TH cell number was most prominent in the center area of substantia nigra pars compacta (SNcc) (Fig. 4B). No neuronal loss was observed in young mice, which did not exhibit motor dysfunction (data not shown). In PD, the reduction in the abundance of TH neurons also occurs primarily in the SNc, consistent with our results. Dopaminergic neuronal loss may contribute to motor impairment observed at the late stages of disease. Furthermore, we tested DA physiology in these 120-week-old *Atg7*^*flox*/*flox*^:TH-Cre mice by neurochemical analysis of the dorsal striata. High-performance liquid chromatography (HPLC) revealed a reduction in striatal DA levels and metabolites in *Atg7*^*flox*/*flox*^:TH-Cre versus control mice (Fig. 4C). Thus, DA content is affected by dopaminergic neuronal loss.

## Discussion

Great progress has been made toward understanding the pathogenesis of familial PD, mainly due to the discovery of specific mutations, whereas little is understood about the mechanisms underlying idiopathic PD pathogenesis. In this study, we carried out detailed observations of *Atg7*-deficient mice, and observed a loss of autophagy in dopaminergic neurons, resulting in Lewy pathology and motor dysfunctions associated with aging. Autophagy maintains the level of endogenous synuclein in DA neurons. In aged *Atg7*^*flox*/*flox*^:TH-Cre mice, synuclein accumulated in the neurites and/or cell bodies involved in inclusions. In addition, these inclusions had characteristics of LBs.

Our results indicate that long-term disruption of autophagy results in widespread synuclein accumulation in DA neurons. In previous report, Friedman *et al*.^9^ reported that dopaminergic axons developed synuclein aggregates at the age of 20 months, and suggested that autophagy is involved in axonal synuclein homeostasis in mice. In our careful observation of *Atg7*^*flox*/*flox*^:TH-Cre mice, we found that the level of endogenous synuclein protein increased at around 9 months in cell bodies and neurites (Fig. 3A,B and C), and these accumulated synuclein deposits contained p62 positive aggregates (Fig. 3D). Our synuclein antibody efficiently detects endogenous mouse synuclein, and a virtual slide system could be used to observe neurons extensively.

In any case, it is clear that autophagy plays a significant role in synuclein degradation in dopaminergic neurons. The protein catabolic pathways that regulate synuclein degradation have been discussed by multiple authors^4,16,17^, and the extent to which autophagy contributes to synuclein degradation depends on the presence of mutations^18^. Several lines of evidence indicate a role for autophagy in the regulation of synuclein homeostasis^5,6,15^, suggesting that dysfunctional autophagic clearance may contribute to the development of synuclein inclusions. Conversely, overexpression of wild-type synuclein impairs autophagic activity, implying a functional relationship between synuclein and autophagic degradation^19^. Furthermore, LB-like synuclein aggregates resist degradation and impair autophagy^20^. In this way, synuclein accumulation may be influenced by the interaction between autophagic impairment and inclusion formation. Intriguingly, in this study we observed for the first time that synuclein deposition is preceded by the formation of p62 inclusion in DA neurons. It is well known that homeostasis of p62 is strictly regulated by autophagy in the brain^10^, and that dysregulation of autophagy causes formation of p62 aggregates. Our results suggest that autophagy regulates synuclein levels to some extent, and we speculate that p62 aggregates caused by autophagic defects serve as ‘seeds’ for synuclein inclusions.

Many questions remain unanswered with respect to LB formation. One hypothesis is that Lewy pathology spreads from the axon to the soma^21^. DA neurons have a unique structural characteristic, long hyper-branched projection axons, that innervate wide areas in the brain^22^. It is likely that this feature increases the chance of developing deposits in peripheral axons and dendrites. In the human brain, synuclein deposition and neuronal degeneration are accentuated in distal regions^23^. In our study, *Atg7*^*flox*/*flox*^:TH-Cre mice exhibited age-related growth and spreading of p62 aggregates in the peripheral region (Fig. 2A). It is likely that aspects of the local environment related to branching may predispose branch regions to synuclein deposition^21^. Our observations that p62- and synuclein-positive inclusions are prone to colocalize in peripheral regions (Fig. 3D and E) support this hypothesis.

Our ultrastructural study revealed that many mitochondria were present in aggregates (Fig. 2Ec,d). During mitochondrial degradation in cultured cells, depolarized mitochondria cluster with p62^24^. Depolarized mitochondria are ubiquitylated by Parkin, recognized by p62, transported via microtubules to aggregates, and degraded by autophagy. Inhibition of physiological microtubule transport and clustering of depolarized mitochondria may contribute to inclusion formation. Electron microscopic analysis by Gai *et al*. revealed that many mitochondria are concentrated in the early forms of LBs^25^, and Bedford *et al*. also reported deposits of mitochondria in early LBs (pale bodies) in PD patients^26^. We speculate that p62 aggregates containing mitochondria (Fig. 2Ec,d) represent inclusions at the early phase of biogenesis of LBs. Thus, Parkin-mediated p62 clustering may be a key process of LB formation, consistent with the fact that LBs are usually absent in autosomal recessive early-onset Parkinsonism caused by Parkin mutations^27^.

None of the current genetic PD models in mouse recapitulates all features of PD. Additionally, only a few of these models develop mild DA neurodegeneration. The most parsimonious explanation for the lack of DA neurodegeneration in genetic PD models is a compensatory mechanism that results from adaptive changes during development, making it hard to observe the degenerative phenotype over the lifespan of mice.

In this study, the age-related motor dysfunction and pathology in mice with *Atg7* deficiency in DA neurons suggests that impairment of autophagy is a potential mechanism underlying the pathology of PD. Our *Atg7*-deficient mice demonstrate typical Lewy pathology, including endogenous synuclein and neuronal loss, which resembles PD. Furthermore DA levels are affected by dopaminergic neuronal loss. Intriguingly, the significant decrease in dopamine is not accompanied by concomitant reductions in the dopamine catabolites DOPAC and HVA. Until now, we did not clarify the reason, but one of the explanation may be presynaptic dysfunction in Atg7-deficient mice^9^. These PD models will provide insight into the process of autophagy in PD pathology, and will be crucial for the development of novel therapeutic targets.

## Materials and Methods

### Animals

All animals were kept in a pathogen- and odor-free environment, which was maintained under a 12 h light/dark cycle at ambient temperature. Procedures were approved by the Animal Experimental Committee of the Juntendo University Graduate School of Medicine, and were performed in accordance with the guidelines of NIH and the Juntendo University Graduate School of Medicine. Floxed *Atg7* mice were characterized previously^8^ and were crossed with TH-Cre mice carrying the knock-in construction containing TH fused to Cre in the 3′ end (gift from Dr. Ted M. Dawson, Johns Hopkins University, USA) to generate *Atg7*^*flox*/*flox*^:TH-Cre mice.

### Behavioral tests

Locomotor behavior was assessed in mice from 90 to 120 weeks of age. Accelerating rotarod tests were performed on a rotarod machine with automatic timers and falling sensors (MK-660D, Muromachi Kikai). Male *Atg7*^*flox*/*flox*^ mice and *Atg7*^*flox*/*flox*^:TH-Cre mice (n = 10 for each genotype) were placed on a 3-cm diameter rotating rod covered with rubber, and rotation was accelerated from 3 to 35 rpm over 5 min. Fall latency was recorded, and the first fall latency of the third trial was used for analysis. The runway test was performed using a narrow, horizontally fixed beam (1 cm wide, 80 cm long, held at a height of 40 cm from the table). The animal was placed at one end of the beam and urged to move toward the opposite end, where an escape platform was located.

### Immunoblot analyses

Anesthetized mice were dissected by decapitation, and lysates for SDS-PAGE were prepared from midbrain tissue as described previously^28^. After electrophoresis, separated proteins were transferred to PVDF membranes (Hybond-P; Amersham Biosciences), which were incubated overnight at 4 °C with the indicated primary antibodies: anti-ubiquitin (Dako), anti-synuclein-1 (BD bioscience), and anti-actin (Millipore: MAB1501). Antibody binding was visualized using the Enhanced Chemiluminescence Kit (GE Healthcare), and signals were detected on an ImageQuant LAS4000 (GE Healthcare).

### Histological analyses

Mice were perfused with 4% paraformaldehyde (PFA), and their brains were immersion-fixed at 4 °C for 36 hrs. The fixed samples were cryoprotected with 20% sucrose and sliced on a freezing microtome to obtain 40-µm-thick floating sections. For double immunohistochemistry of p62 and TH, sections were initially incubated with the anti-p62 antibody (PROGEN Biotechnik, GmbH) and visualized with diaminobenzidine (DAB)-containing nickel ammonium sulfate (DABNi), which generates dark purple precipitates. The same section was then incubated with the anti-TH antibody (657012, Calbiochem, Germany), and visualized with DAB, which generates brown precipitates. The sections were observed on a VS120 (Olympus, Tokyo, Japan). For double immunofluorescence of p62 (green) and TH (red), floating sections were incubated with rabbit anti-TH antibody (657012, Calbiochem, Germany) and guinea pig anti-p62 antibody (PROGEN Biotechnik, GmbH). They were then incubated with anti–guinea pig IgG conjugated with Alexa Fluor 488 and anti-rabbit IgG conjugated with Alexa Fluor 546. For double immunofluorescence study of synuclein (green) and p62 (red), floating sections were incubated with the rabbit anti-synuclein antibody (AB5038P, Millipore, USA) and guinea pig anti-p62 antibody (PROGEN Biotechnik, GmbH). They were then incubated with anti-rabbit IgG conjugated with Alexa Fluor 488 and anti–guinea pig IgG conjugated with Alexa Fluor 546. Fluorescent signals were captured by LSM 780 confocal microscopy (Zeiss).

### Electron microscopy and immunoelectron microscopy with DAB

For conventional electron microscopy, mice were fixed by cardiac perfusion with 2% PFA and 2% glutaraldehyde in 0.1 mol/L PB (pH 7.2). Brain slices were embedded in epoxy resin, and ultrathin sections (70 nm thick) were cut and observed on a Hitachi HT7700 electron microscope (Hitachi, Tokyo, Japan). For immunoelectron microscopy with DAB, mice were perfused and fixed with 4% paraformaldehyde, 0.5% glutaraldehyde (GLA), and 15% saturated picric acid in 0.1 M phosphate buffer (PB) for 1 hr at room temperature (RT). Coronal 50 µm-thick slices of substantia nigra (SN) were prepared with a microslicer (VT1200S, Leica Microsystems, GmbH), and SN-containing slices were subjected to the indicated immunohistochemical analysis. Slices were treated with 0.6% hydrogen peroxide in saline for 15 mins. After washing, the slices were incubated for 6 weeks at 4 °C with guinea pig anti-p62 antibody (PROGEN Biotechnik, GmbH) in 1% bovine serum albumin in 0.1 M phosphate-buffered saline (PBS). Subsequently, the slices were incubated for 2 hrs at RT with horseradish peroxidase–conjugated anti–guinea pig IgG antibody (Jackson ImmunoResearch, USA), and then developed with DABNi. The immunoreacted slices were re-fixed with 2.5% GLA in 0.1 M cacodylate buffer (CB) for 15 min at 4 °C. After several rinses in 0.1 M CB with 4.5% sucrose, the slices were post-fixed with 2% osmium tetroxide in 0.1 M CB for 2 hrs at 4 °C, dehydrated in a graded series of ethanol followed by propylene oxide, and then horizontally embedded in epoxy resin (EPON 812, TAAB, UK). Embedded samples were sectioned into 4 µm-thick serial semi-thin sections, and identified based on DABNi color development under a light microscope. Subsequently, sections that contained target lesions were adhered onto new blocks of epoxy resin. Ultrathin sections for silver and/or gray interference color were cut from the region, placed in formvar-coated one-slot grids, stained with uranyl acetate and lead citrate, and examined on a transmission electron microscope (JEM-1400, JEOL, Tokyo).

### HPLC Analysis

Dorsal striata from 120-week-old mice were dissected, quickly frozen on dry ice, and then homogenized with 0.5 mL of 0.2 M perchloric acid containing 100 µM EDTA-2Na per 100 mg wet tissue. Samples were centrifuged at 20,000 × g for 15 min at 4 °C. The supernatant was collected and analyzed by HPLC.

### Statistical analysis

Statistical significance was determined by Student’s t-test (STATVIEW; SAS Institute). Data are expressed as means ± S.E. P < 0.05 was considered significant.

## Electronic supplementary material

Supplemental Info
