# Supplementary material for: Loss of autophagy in dopaminergic neurons causes Lewy pathology and motor dysfunction in aged mice
Source: Sci Rep. 2018 Feb 12;8:2813. doi: 10.1038/s41598-018-21325-w (PMC5809579; doi:10.1038/s41598-018-21325-w)
Supplement: Supplementary file 1 — Supplemental Info [file 41598_2018_21325_MOESM1_ESM.pdf]

Loss of autophagy in dopaminergic neurons causes Lewy pathology and motor dysfunction in aged mice

Shigeto Sato[1]*, Toshiki Uchihara[2], Takahiro Fukuda[3], Sachiko Noda[1], Hiromi Kondo[4], Shinji Saiki[1], Masaaki Komatsu[5], Yasuo Uchiyama[6], Keiji Tanaka[7], Nobutaka Hattori[1]*

Supplemental Figure legends

Supple. Figure 1. The Atg7 reduction and specific synuclein accumulation

(A) Immunostaining with Atg7 in LC (a, d) and cerebellum (c, f) of 12-month-old *Atg7*$^{flox/flox}$:TH-Cre and *Atg7*$^{flox/flox}$ mice. Immunostaining with TH in LC (b, e) using serial section. Scale bars: 20 μm. (B) Representative midbrain (left panel) and cerebellum (right panel) immunoblot of endogenous Atg7 in 12- month-old *Atg7*$^{flox/flox}$ mouse (left lane) and *Atg7*$^{flox/flox}$:TH-Cre mouse (right lane). Quantitation of the immunoblot for Atg7; ratio of Atg7 to actin is shown. Atg7 are remarkably decreased in the midbrains of Atg7$^{flox/flox}$:TH-Cre mice. Data show means ± SE (n = 3). ** $P < 0.01$ (Student's t-test). (C) Immunoblot of synuclein in 12-month-old *Atg7*$^{flox/flox}$:TH-Cre mouse (lanes 1-3: cerebellum; lanes 4-6: midbrain) reveals tissue specific synuclein

accumulation.

Supple. Figure 2. P62 and synuclein inclusions were present along the TH fibers.

(A) Histological analyses of substantia nigra (SN) in a 9-month-old *Atg7*$^{flox/flox}$ mouse. Thickly sliced (40-µm) cryosections were immunostained for synuclein, and the SN was broadly observed on a VS120 microscopic 3D measurement system (Olympus). Bar: 100 µm. (B) Low magnification image of histological analyses of dopaminergic neurons in an 18-month-old *Atg7*$^{flox/flox}$:TH-Cre mouse. Cryosections were immunostained for p62 (black) and TH (brown) Scale bars: 100 µm. Square area is enlarged in Fig. 2C a. (C) High resolution confocal images through SN show immunofluorescence labeling of TH (red) and synuclein (green), where yellow indicates synuclein within TH neurons. Scale bars: 40 µm.

Supple. Figure 3. Time-dependent synuclein accumulation

(A) Representative time-dependent immunoblot of endogenous synuclein in 3- (lane 1), 18- (lane 2), and 30- (lane 3) month-old *Atg7*$^{flox/flox}$ mice and 3- (lane 4), 18- (lane 5),

and 30- (lane 6) month-old *Atg7*$^{flox/flox}$:TH-Cre mice.

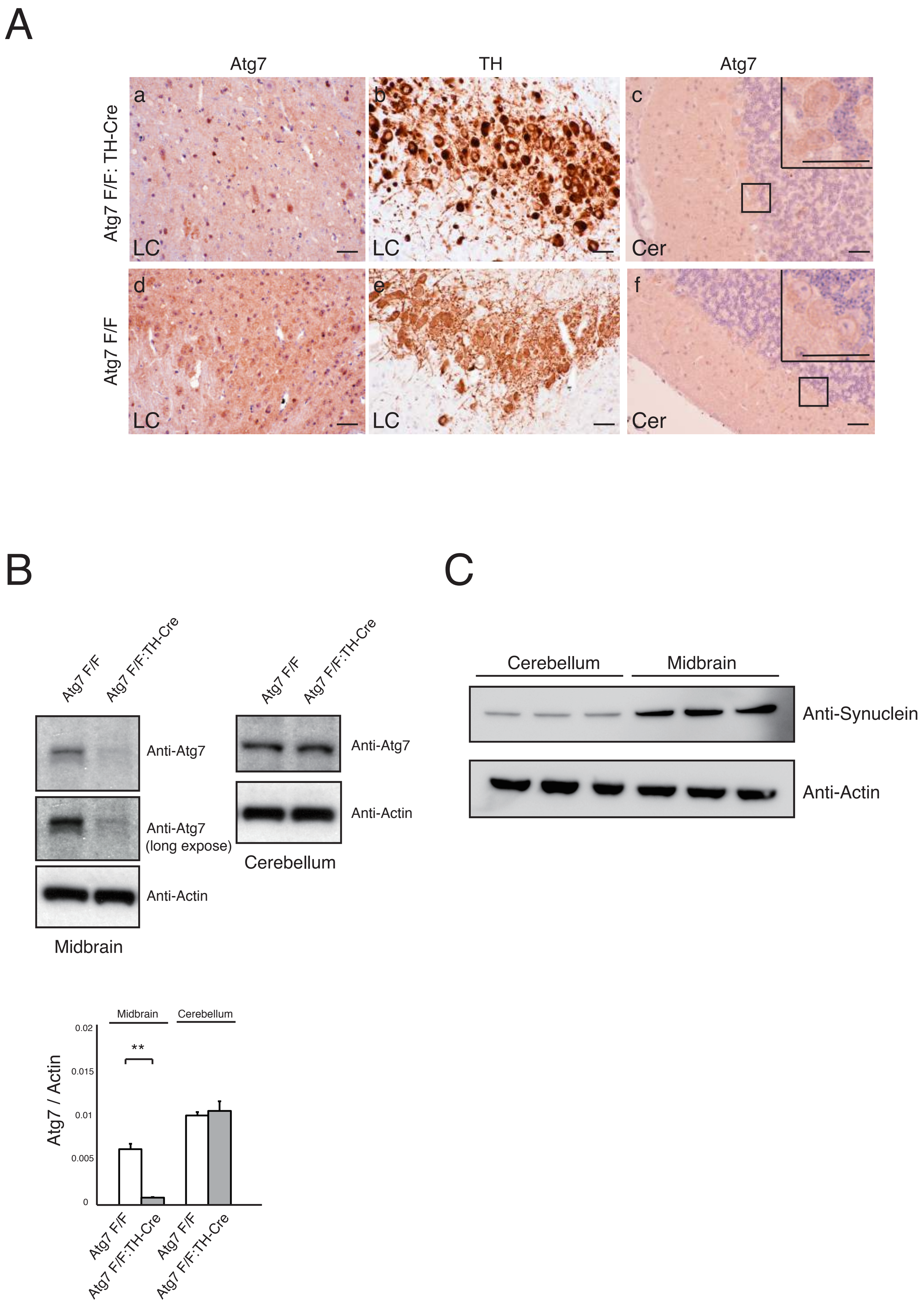

Supple. Figure 1. The Atg7 reduction and specific synuclein accumulation

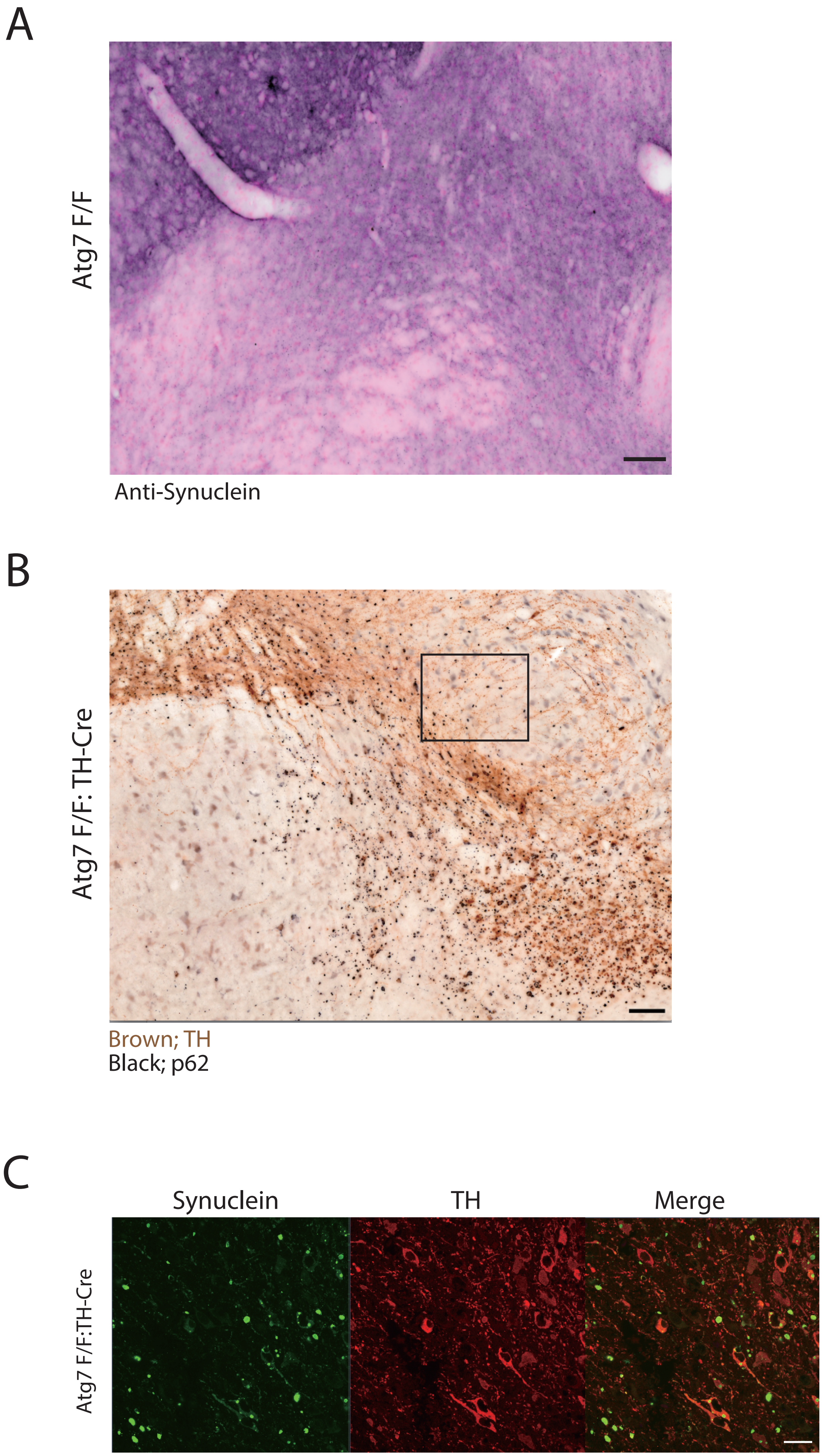

Supple. Figure 2. P62 and synuclein inclusions were present along the TH fibers.

A

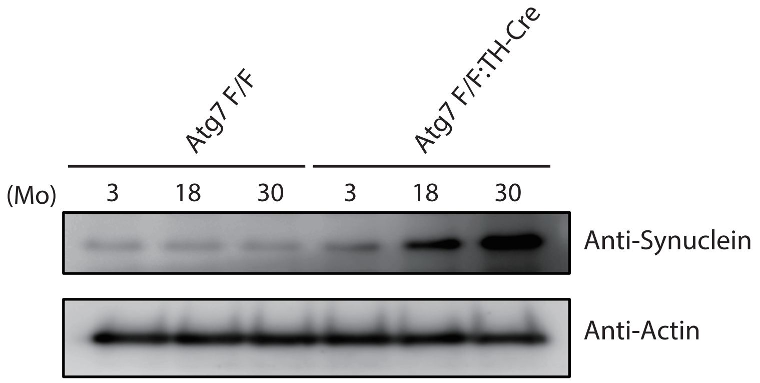

Supple. Figure 3. Time-dependent synuclein accumulation
